# Supplementary material for: Simulating the human colorectal cancer microenvironment in 3D tumor-stroma co-cultures in vitro and in vivo
Source: Sci Rep. 2020 Jun 17;10:9832. doi: 10.1038/s41598-020-66785-1 (PMC7300090; doi:10.1038/s41598-020-66785-1)
Supplement: Supplementary file 1 — Supplementary Information. [file 41598_2020_66785_MOESM1_ESM.docx]

**Simulating the human colorectal cancer microenvironment in 3D tumor-stroma co-cultures *in vitro* and *in vivo***

Mahesh Devarasetty^1^, Anthony Dominijanni^1^, Samuel Herberg^2^, Ethan Shelkey^1^, Aleksander Skardal^3^, Shay Soker^1,^*

**^1^** Wake Forest Baptist Medical Center; Winston-Salem, NC 27101

^2^ SUNY Upstate Medical University; Syracuse, NY 13210

^3^ The Ohio State University; Columbus, OH 43210

*Primary correspondence to:

Shay Soker

391 Technology Way

Winston-Salem, NC 27101

ssoker@wakehealth.edu

**

**

**Supplementary Figure 1: Construct fabrication and gross morphology.** (**A**) Molds were produced through additive printing of a negative mold, then deposited silicone was cured to yield a 6-well plate microwell insert. Then cell-hydrogel solution was deposited, with a suspended HCT-116 spheroid, into microwells and allowed to gelate, then self-assemble over experimental timeline before being harvested for further analysis. (**B**) Images of constructs after 1 day in culture. The magnitude of LX2 co-cultures size contraction compared to collagen-only controls can be appreciated.

**
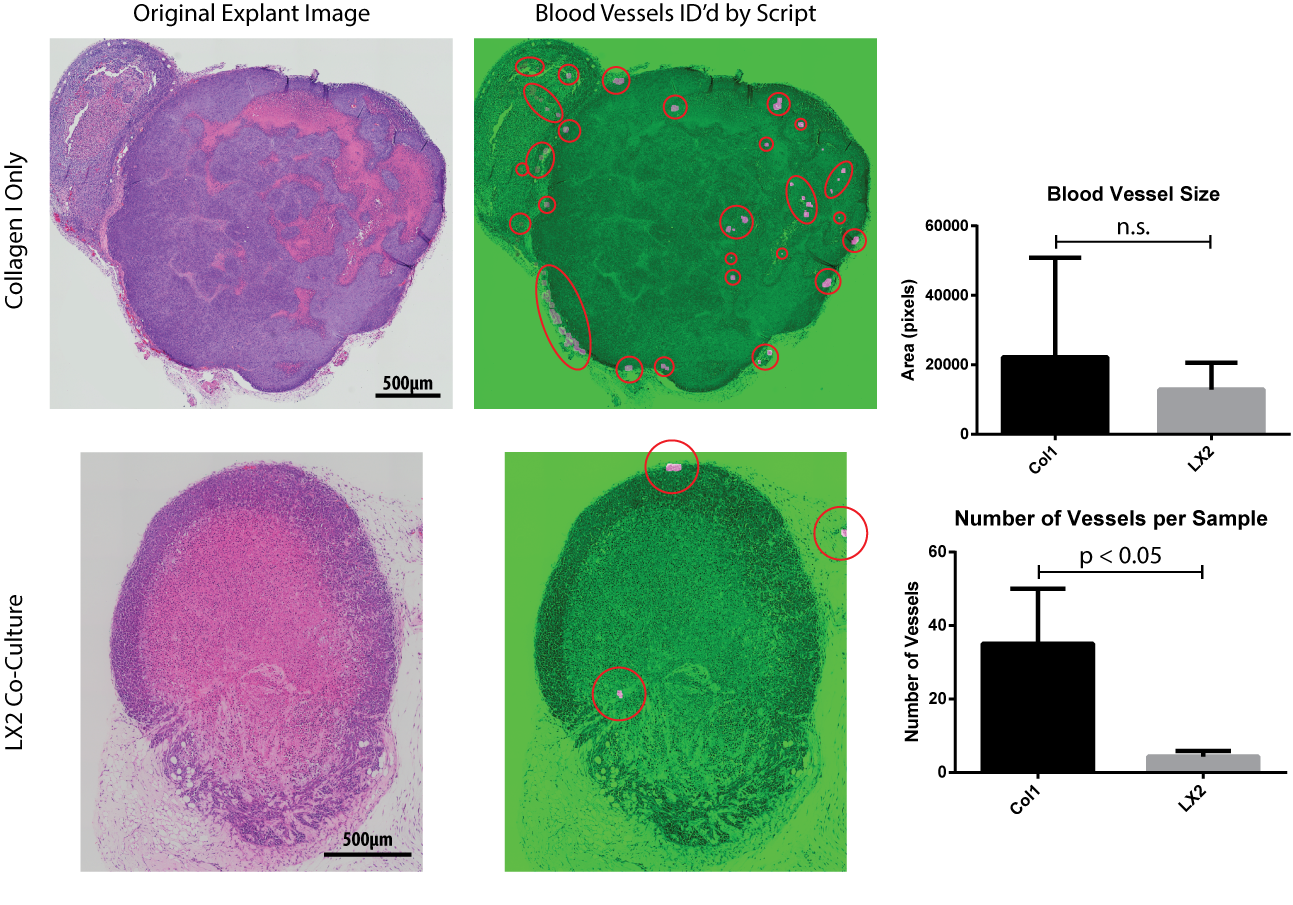
**

**Supplementary Figure 2: Blood vessel quantification.** H&E-stained micrographs (left) were used for analysis of blood vessel quantity and size. Vessels appear as a specific red hue which can be segmented efficiently. A MatLab script is used to identify vessel structures, then size and number of vessels were quantified (right). Collagen I only stroma induce the formation of more vessels, but vessel size remains consistent compared to LX2 co-cultures. Refer to supplemental file vesselQuant.m for script.

**
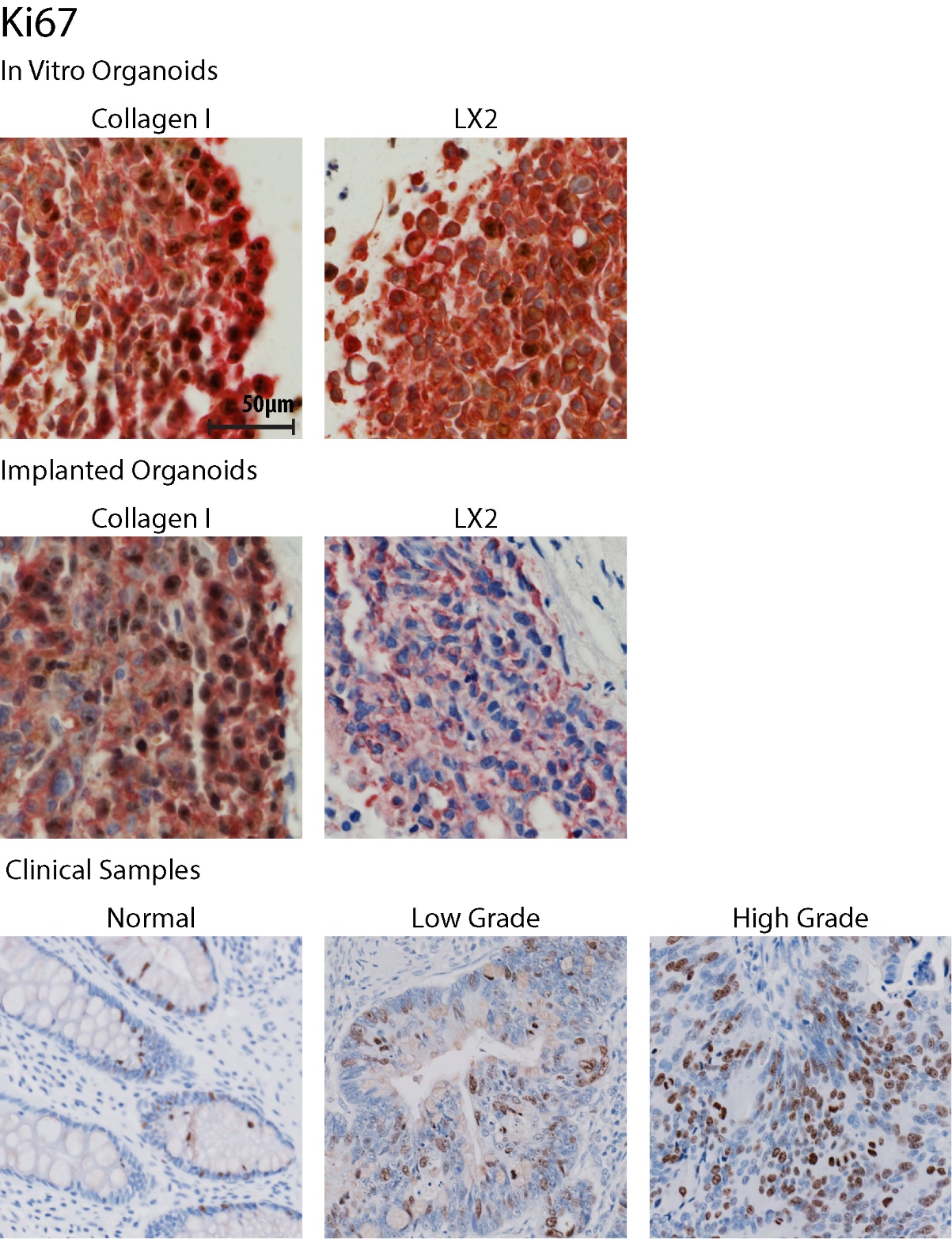
**

**Supplementary Figure 3: Ki67 immunohistochemical staining.** Both *in vitro* and *in vivo* samples were double stained (DAB and Vector Red) to identify HCT-116 cells from LX2 and mouse cells. DAB labels Ki67 and Vector Red labels CK18 which is specific to the HCT-116 cells in this system. Clinical samples were stained for Ki67 only, with DAB as the chromogenic label.

**
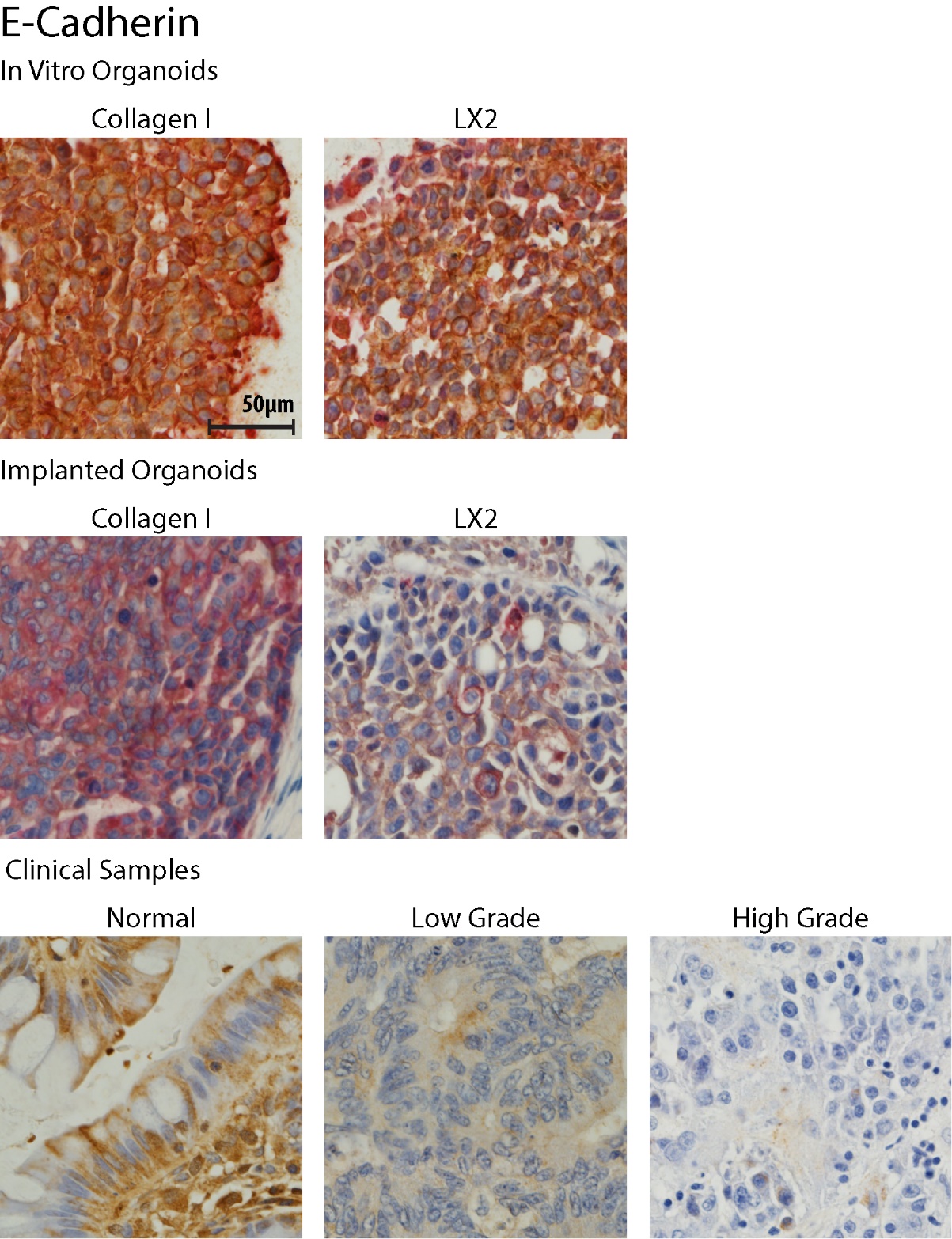
**

**Supplementary Figure 4: E-Cadherin immunohistochemical staining.** Both *in vitro* and *in vivo* samples were double stained (DAB and Vector Red) to identify HCT-116 cells from LX2 and mouse cells. DAB labels E-Cadherin and Vector Red labels CK18 which is specific to the HCT-116 cells in this system. Clinical samples were stained for E-Cadherin only, with DAB as the chromogenic label.

**
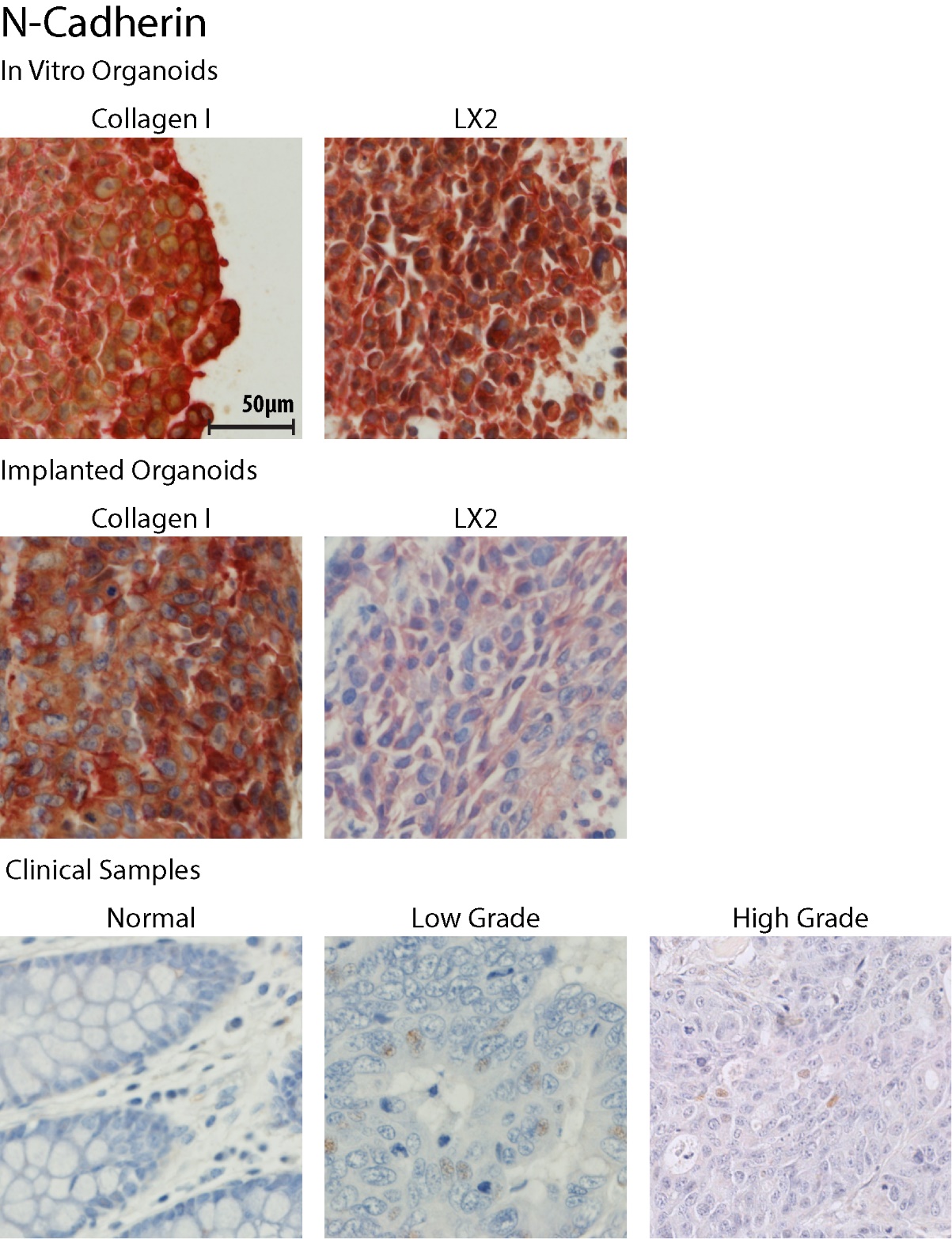
**

**Supplementary Figure 5: N-Cadherin immunohistochemical staining.** Both *in vitro* and *in vivo* samples were double stained (DAB and Vector Red) to identify HCT-116 cells from LX2 and mouse cells. DAB labels N-Cadherin and Vector Red labels CK18 which is specific to the HCT-116 cells in this system. Clinical samples were stained for N-Cadherin only, with DAB as the chromogenic label.

**
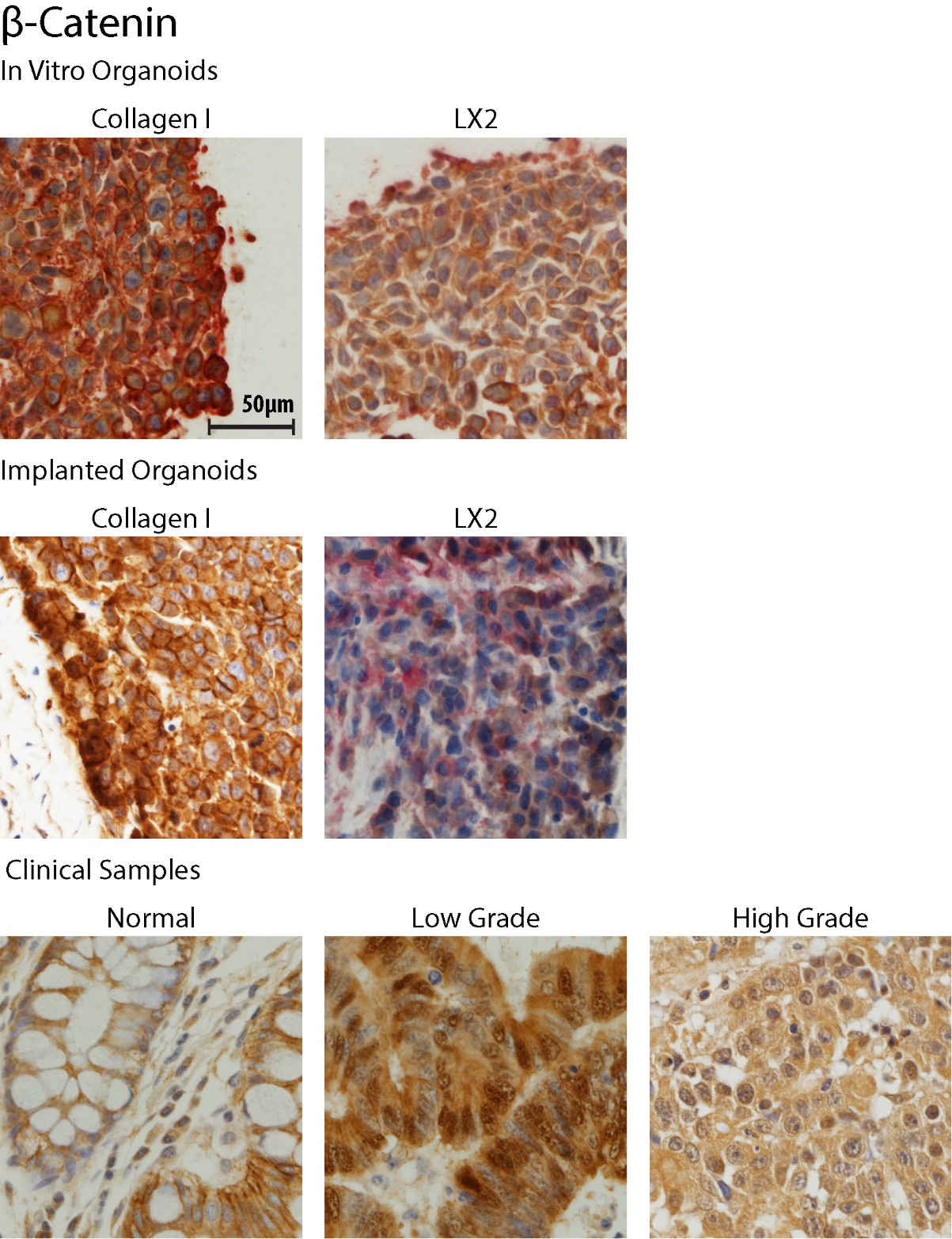
**

**Supplementary Figure 6: β-Catenin immunohistochemical staining.** Both *in vitro* and *in vivo* samples were double stained (DAB and Vector Red) to identify HCT-116 cells from LX2 and mouse cells. DAB labels β-Catenin and Vector Red labels CK18 which is specific to the HCT-116 cells in this system. Clinical samples were stained for β-Catenin only, with DAB as the chromogenic label.

**
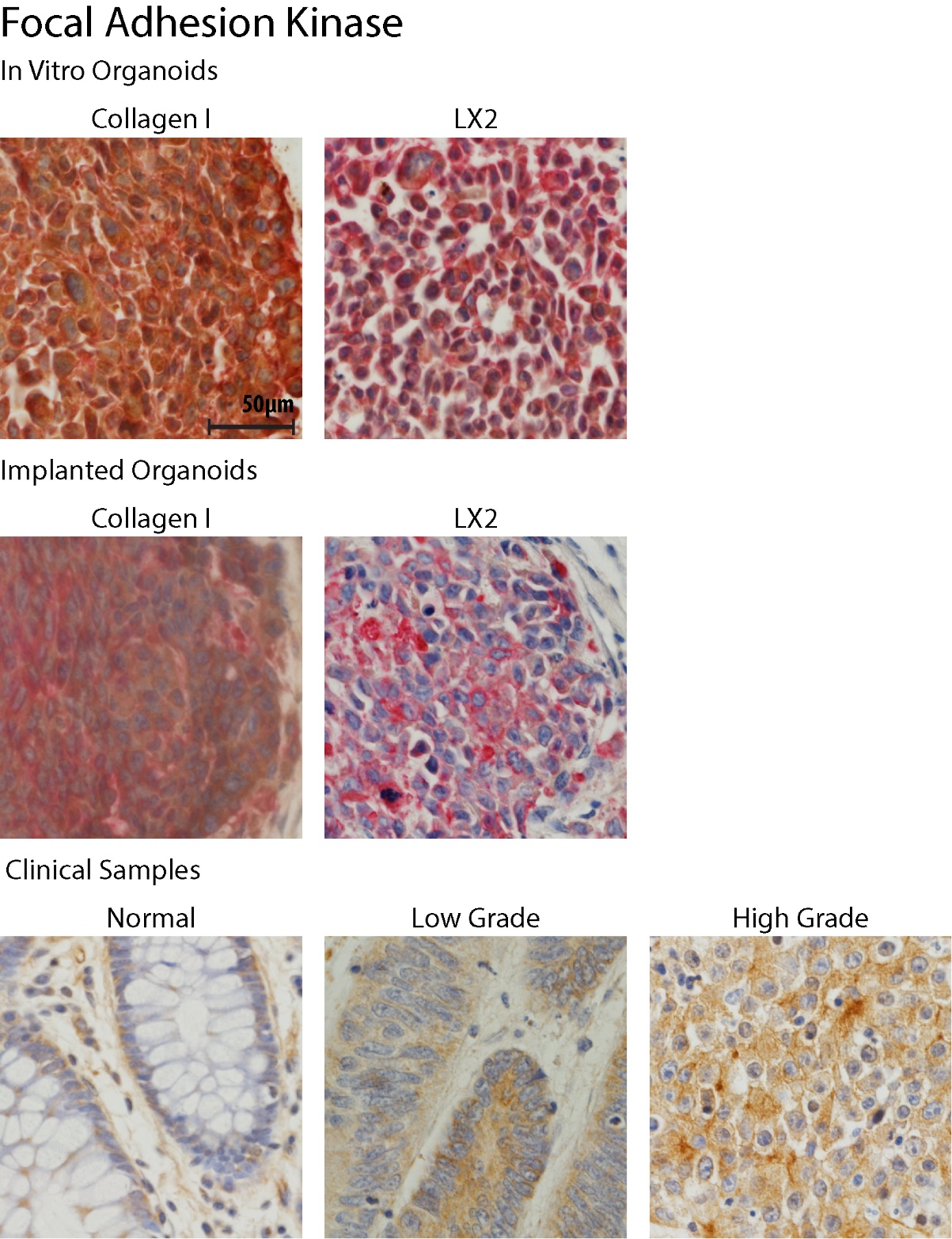
**

**Supplementary Figure 7: FAK immunohistochemical staining.** Both *in vitro* and *in vivo* samples were double stained (DAB and Vector Red) to identify HCT-116 cells from LX2 and mouse cells. DAB labels FAK and Vector Red labels CK18 which is specific to the HCT-116 cells in this system. Clinical samples were stained for FAK only, with DAB as the chromogenic label.

**
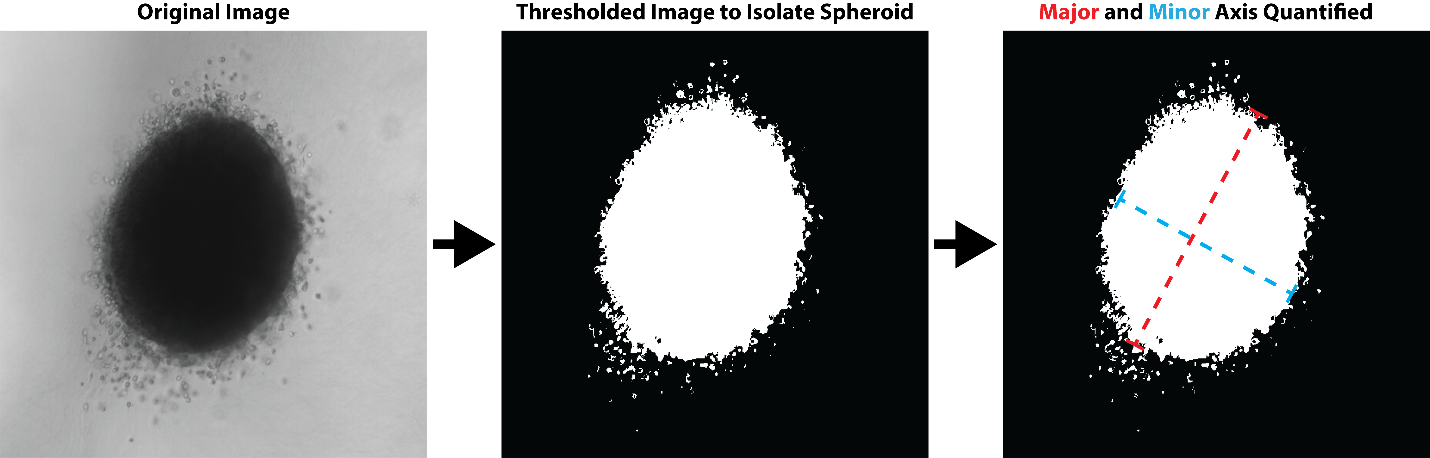
**

**Supplementary Figure 8: Quantification of spheroid size after construct culture.** Samples were imaged under brightfield conditions to visualize the spheroid body. Images were then captured at various z-levels and combined to generate final images. A MatLab script was then used to segment the spheroid from surrounding space and quantify average diameter using major and minor axes. Refer to supplemental file areaQuant.m for script.

**
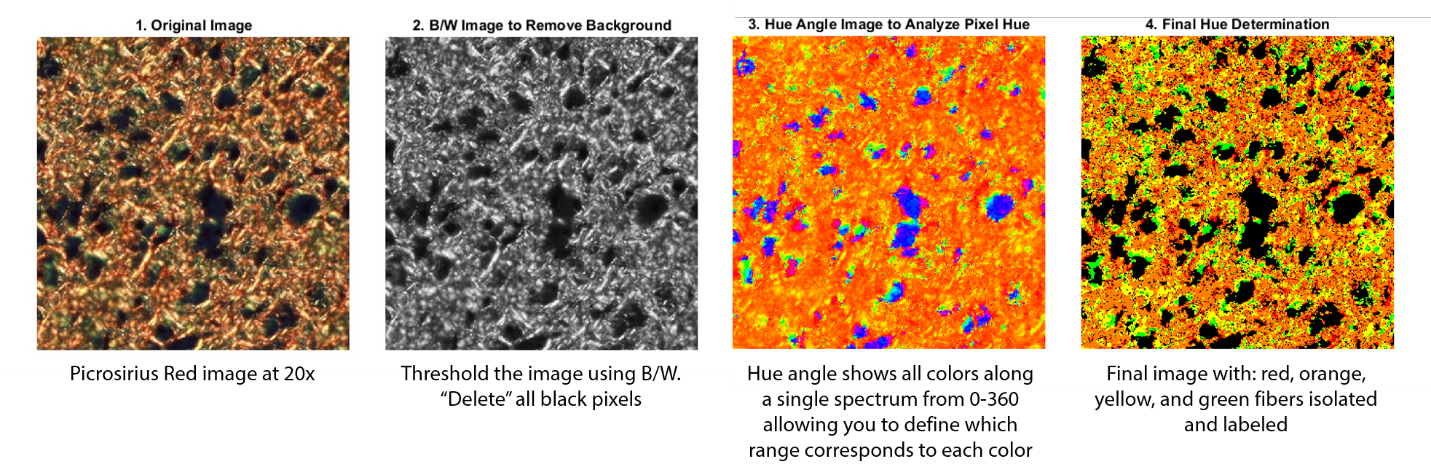
**

**Supplementary Figure 9: Picrosirius red hue analysis.** PSR-stained images were captured under polarized light, then a MatLab script was used to quantify pixels of varying hue signal. Briefly, the workflow for this process is: 1) import the image into Matlab, 2) generate a black and white image to find and ‘delete’ all empty space (black pixels) from the original image, 3) convert the background subtracted image to an HSV format which identifies all pixel hues along a single channel spectrum from 0-360, 4) using pre-generated thresholds for hue, segment each pixel into red, orange, yellow, and green while excluding all other colors. This final image can be quantified by counting pixels of each segmented classification. Refer to supplemental file hueQuant.m for script.

**
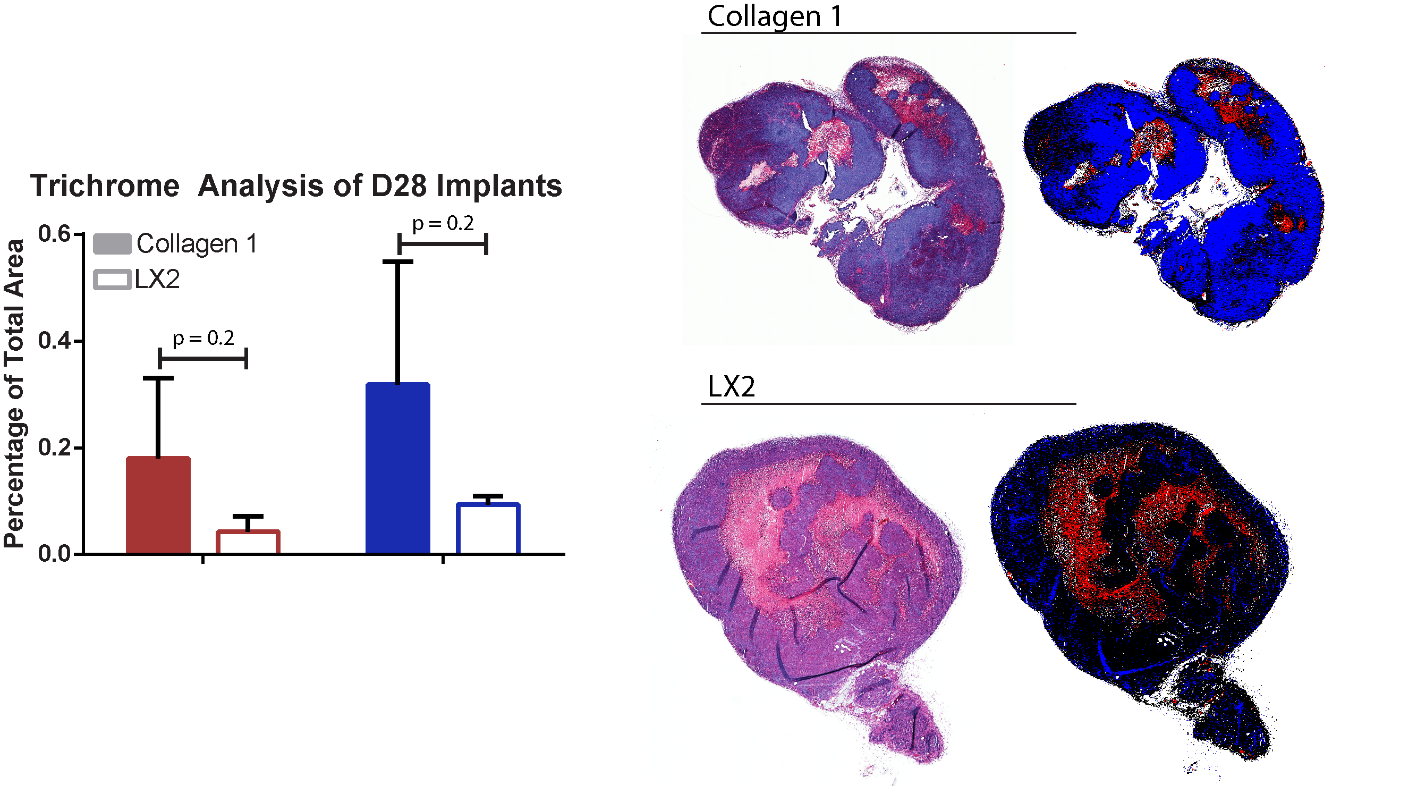
**

**Supplementary Figure 10: Trichrome staining and analysis.** To assess the collagen content of long-term implants, *In vivo* implants were explanted at day 28 then stained with Masson’s Trichrome and bright field images were analyzed using a MatLab script to segment blue and red pixels. Blue stain localizes specifically to collagen and red stain localizes specifically to areas of connective tissue or other ECM proteins. Refer to supplemental file trichQuant.m for script.


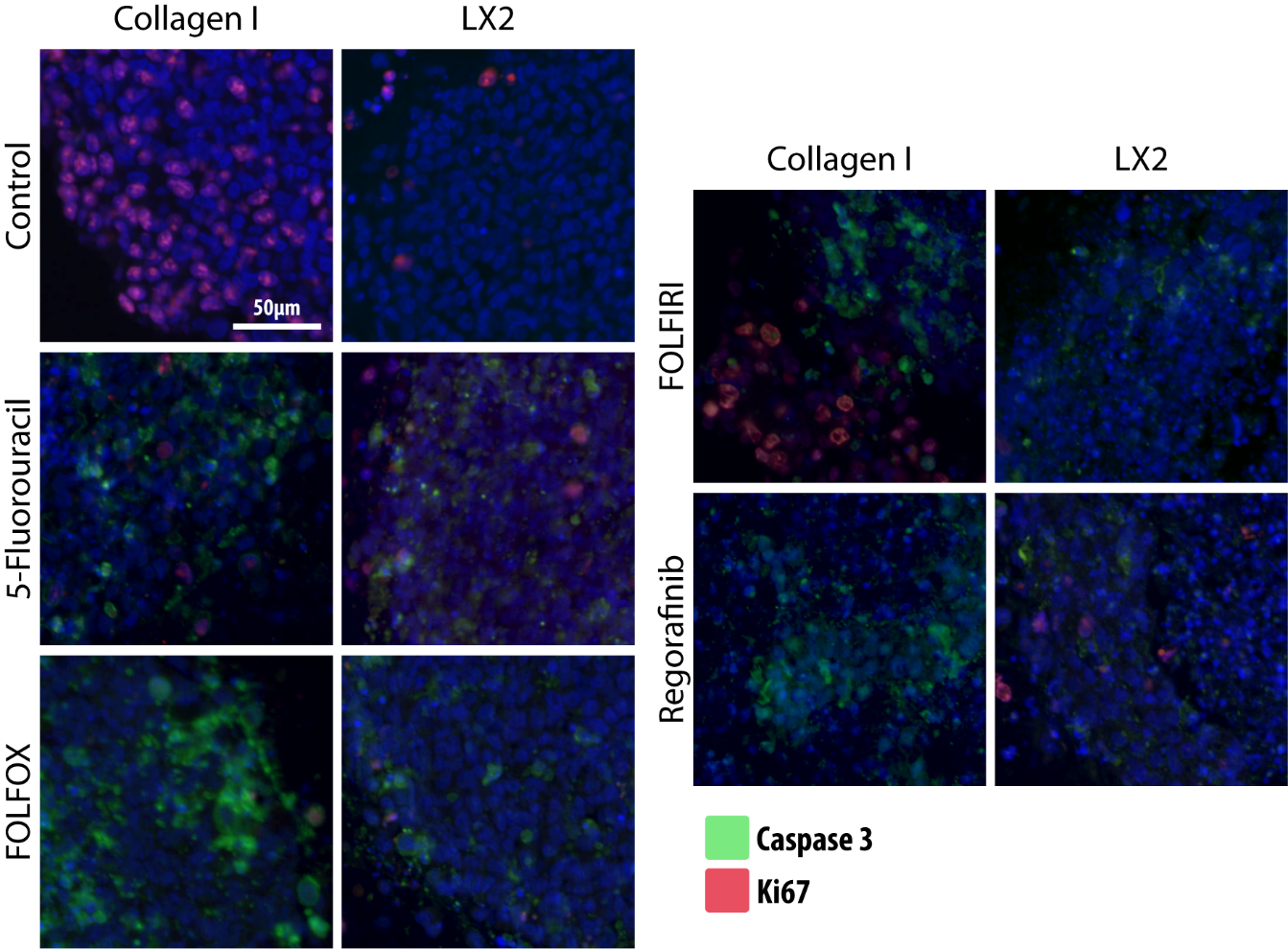


**Supplementary Figure 11: Caspase 3 and Ki67 IHC of chemotherapy treated constructs.** Samples were treated with various combinations of common chemotherapeutic regimen to assess changes in susceptibility between those cultured in collagen-only or LX2-organized conditions. These images are representative of the dataset utilized for automatic quantification with Visiopharm. Caspase 3 (green) staining specifically identifies apoptotic cells with membrane/cytoplasmic labeling. Ki67 (red) specifically identifies proliferating cells with nuclear labeling.


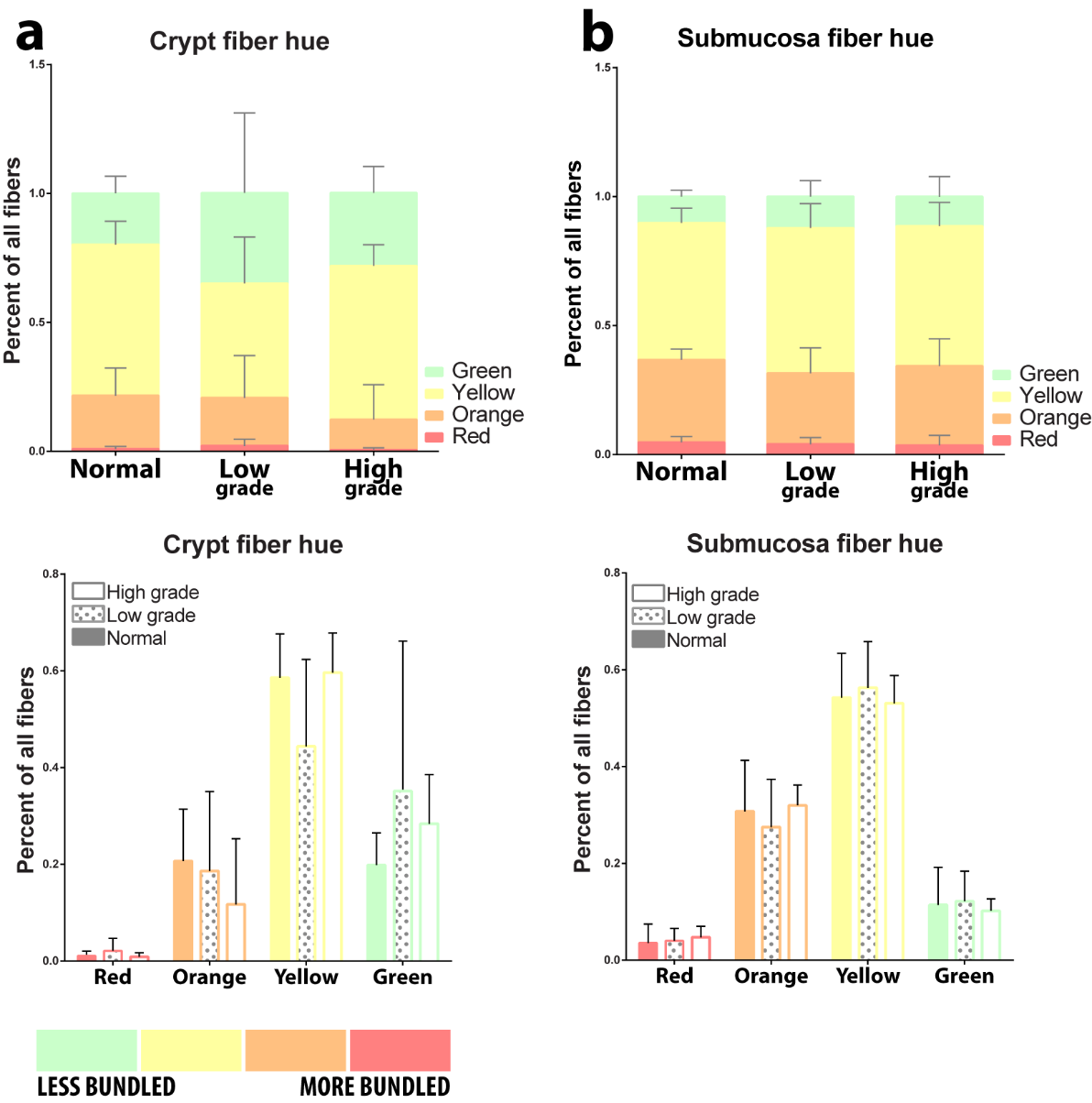


**Supplementary Figure 12: Collagen fiber hue analysis of clinical specimen.** Polarized images of picrosirius red stained clinical samples were analyzed using a custom Matlab script to extract information about fiber color (outlined in **Suppl. Fig. 9**). Green and yellow correspond to reticular fibers while orange and red correspond to thick bundles. Crypt (a) fibers and submucosal (b) fibers were analyzed separately as they show distinct architectural features. Analysis of fiber hue describes the collagen network maturation. In these clinical samples, there were minor differences in hue between healthy and tumor samples indicating collagen turnover and reorganization during tumorigenesis does not significantly impact overall fiber maturity. This result indicates that existing fibers may be moved or restructured to produce cancerous ECM architecture rather than be generated from newly deposited collagen I.





**Supplementary Figure 13: Visiopharm analysis pipeline.** Triple stained images (Hematoxylin, DAB, and Vector Red) are captured on an upright, light microscope (a). Then deconvolutions were performed to generate separate channels of each stained pattern. These deconvolutions accurately and consistently separate the chromogenic signals from each other for isolated analysis (b). With each separate channel, masks are produced around each nuclei (from hematoxylin channel) and labeled for positive DAB or Vector Red to produce the final labeled image (c). The labels can be quantified within Visiopharm for comparison and analysis.
